# Sensitivity Enhancement of 2D Material-Based Surface Plasmon Resonance Sensor with an Al–Ni Bimetallic Structure

**DOI:** 10.3390/s23031714

**Published:** 2023-02-03

**Authors:** Miaosen Hu, Min Li, Ming-Yu Li, Xiaoyan Wen, Shuo Deng, Sisi Liu, Haifei Lu

**Affiliations:** 1 School of Science, Wuhan University of Technology, Wuhan 430070, China; 2 Yangtzi Delta Region Institute of University of Electronic Science and Technology of China, Huzhou 313098, China

**Keywords:** surface plasmon resonance, 2D materials, lateral position shift, angular sensitivity

## Abstract

In this paper, a variety of 2D materials on the surface plasmon resonance sensor based on Al–Ni bimetallic layer are compared. Simulation results indicate that lateral position shift, which is calculated according to the real and imaginary parts of the refractive index of material, can be used as an effective parameter to optimize the sensitivity. By using the parameters for optimizing the SPR structures, the results show that the multiple layer models of Al(40 nm)–Ni(22 nm)–black phosphorus (BP)(1 L) and Al(40 nm)–Ni(22 nm)–blue phosphorus (BlueP)/WS_2_(1 L) exhibit average angular sensitivities of 507.0 °/RIU and 466 °/RIU in the refractive index range of 1.330–1.335, and maximum sensitivity of 542 °/RIU and 489 °/RIU at the refractive index of 1.333, respectively. We expect more applications can be explored based on the highly sensitive SPR sensor in different fields of optical sensing.

## 1. Introduction

Surface Plasmon Resonance (SPR) is a powerful technology used to detect the refractive index variation of sensing medium due to the local electric field with high intensity at the interface of metal and sensing medium. Due to its high precision, high sensitivity, and fast detection ability, SPR sensors have been widely explored in gas sensing [1], biochemical sensing [2,3] and food safety [4]. The conventional structure based on SPR is set up on a Kretschmann structure with a glass prism for phase matching and excitation of surface plasmon polariton (SPP) on the noble metal film, such as gold (Au) [5,6] and silver (Ag) [7,8]. However, the SPR sensor based on a single-layer metal film has low sensitivity, which is limited by the absorption loss of the metal layer. Therefore, the bimetallic layer has been proposed to improve the sensitivity of SPR sensors [9,10]. Yun et al. designed an SPR sensor based on Ag–Au bimetallic layer, which demonstrated a sensitivity improvement of 15% compared with the conventional SPR sensor based on the single metal film [11]. Compared to the normally used metal of Au and Ag, alumina (Al) had been demonstrated to show even higher sensitivity in the multi-layer sensitized SPR structure due to its efficient transition of light energy to surface plasmon [12]. In recent years, ferromagnetic nickel (Ni) has attracted attention because of its inexpensive price [13,14], and the SPR sensor using a single layer of Ni had demonstrated a sensitivity of 168 °/RIU [15]. The even higher sensitivity of the SPR sensor based on the bimetallic layer of Cu and Ni had been proposed by Vibisha et al. [16]. They showed that the sensitivity of 480 °/RIU can be achieved for the configuration of Cu–Ni–WS_2_.

Recently, 2D materials such as graphene [17,18,19,20] and transition metal dichalcogenides (TMDC) [21,22,23] have attracted wide interest due to their unique structural and optical properties. The unique 2D structure and large specific surface area make the sensor based on 2D materials an excellent application prospect in the biochemical sensing area [24,25]. Meanwhile, the stable chemical property makes 2D materials widely used as the outermost layer of the SPR sensor to protect the internal chemical unstable structure [26,27]. Ouyang et al. suggested a new configuration of SPR sensor with TMDC and showed that the sensitivity of 155.68 °/RIU can be achieved for the configuration of the Au–silicon–WS_2_ monolayer [28]. Recently, black phosphorus (BP) has received extensive attention due to its low absorption, and the heterostructure of BP and bilayer WSe_2_ on the BK7 prism can improve the sensitivity to 279 °/RIU [29]. In addition, the novel 2D material of blue phosphorus (BlueP)/TMDC with similar hexagonal crystal-like structure has attracted attention [30]. Shivangani et al. theoretically presented a SPR sensor by using BlueP/WS_2_ on Ag–Al_2_O_3_–Ni and exhibited the sensitivity of 374 °/RIU at 633 nm [31].

To understand the contribution of 2D materials to the sensitivity improvement of SPR structure, some studies have suggested that the large real part of the dielectric constant of the 2D material layer has accounted for enhancing the local electric field between the 2D material and the external medium, while the imaginary part is only explained as energy loss [32]. Other studies believe that the absorption of 2D materials improves the incident light energy transferring to SPP, and thus enhances the sensitivity of the sensor. These conclusions are mainly focused on the optimization of the 2D material layer number [33]. The comprehensive study on the influence of 2D materials in the SPR sensor with multi-layer structure is still necessary.

In this paper, a new SPR structure consisting of Al–Ni bimetallic layer and 2D material (i.e., TMDC, BP, BlueP) has been proposed. The minimum reflectance, full width at half-maximum (FWHM) of resonance curve, sensitivity, and figure of merit (FOM) of the SPR sensors are studied by using the transmission matrix method (TMM) and Fresnel formula. The calculation results show that the maximum sensitivities of 489 °/RIU and 542 °/RIU have been achieved for Al–Ni–BlueP/WS_2_(1 L) and Al–Ni–BP(1 L), respectively. More importantly, the influence of the dielectric constant of the outermost 2D material on the sensitivity of the SPR sensor has been investigated. Based on our calculations, both real and imaginary parts of the 2D material refractive index affect the sensitivity of the SPR sensor. Thus, the lateral position shift considering both the real part and the imaginary part of the refractive index has been proposed as the appropriate physical parameter for the optimization of 2D materials, which has also been demonstrated to be consistent with the results based on the surface electric field intensity.

## 2. Structure and Numerical Model of SPR Sensor

### 2.1. Refractive Index of Various Layer Components

**Figure 1:**
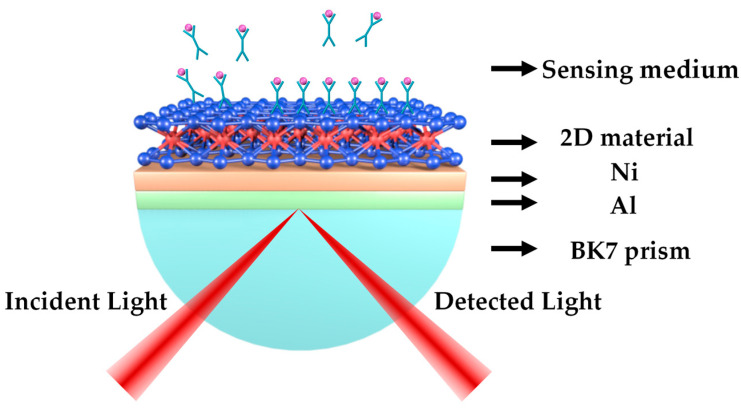
Schematic diagram of the proposed SPR sensor.

SPR sensor based on Kretschmann configuration comprised of BK7 prism, Al, Ni, 2D material, and external sensing medium is shown in Figure 1. The p-polarized light working at the wavelength of 633 nm is incident on the metal surface through the BK7 prism for the excitation of SPP at the 2D material/sensing medium interface, and the refractive index change of the sensing medium can be recorded by monitoring the reflected light with a coaxially reversed photodetector. The response and sensitivity of the SPR sensor are discussed by the angular interrogation method in this paper. The refractive index (RI) of the BK7 prism is calculated to be 1.5151 utilizing the equation as follows [23]:

(1) $$n_{BK7}={\left(\frac{1.03961212\lambda^{2}}{\lambda^{2}-0.0060006986}+\frac{0.231792344\lambda^{2}}{\lambda^{2}-0.0200179144}+\frac{1.01046945\lambda^{2}}{\lambda^{2}-103.560653}+1\right)}^{1/2}$$

The metal layer Al is selected to excite SPP instead of Au and Ag. Compared with noble metals, Al is chemically unstable and will be easily oxidized in practical applications. Here, the nickel (Ni) layer is selected as the second metal layer for simultaneously improving the sensitivity of the sensor and protecting the Al layer from oxidation. The dielectric constants of the two metal layers are calculated by the Drude model and given by [12,15]:

(2) $$n_{\mathrm{metal}}=\left[1-\frac{\lambda^{2}\lambda_{c}}{\lambda_{p}^{2}\left(\lambda_{c}+i\lambda \right)}\right]$$

where *λ_p_* = 1.0657 × 10^−7^ m and *λ_c_* = 2.4511 × 10^−5^ m represent the wavelength of plasma and collision of Al, respectively. For Ni, *λ_p_* =2.5381 × 10^−7^ m and *λ_c_* = 2.8409 × 10^−5^ m.

Due to the unique features of 2D materials which are suitable as an affinity layer for the analyte and improve the sensitivity of the SPR sensor, several 2D materials, e.g., MoS_2_, WS_2_, MoSe_2_, WSe_2_, BP, and BlueP are adopted to cover the bimetallic layer for the study of refractive index sensing performance. The refractive index at the wavelength of 633 nm and monolayer thickness of all 2D materials used in the paper are listed in Table 1 [29,34,35]. Finally, the outermost layer is the sensing medium, and its refractive index is assumed to vary in the range of 1.330~1.336.

### 2.2. Theoretical Model of SPR Sensor and Evaluation Parameters

We employed the transfer matrix method (TMM) to analyze the electromagnetic fields of the SPR structure with the multilayer. The relationship between the electric/tangential field of each layer and the first layer can be expressed as:

(3) $$\left[\frac{E_{1}}{H_{1}}\right]=M\left[\frac{E_{N-1}}{H_{N-1}}\right]$$

where *E*_1_, *E*_N–1_, *H*_1_, and *H*_N–1_ are the tangential components of the electromagnetic field at the first and $N^{th}$ layer boundary. The characteristic matrix (M) of the multi-layer structure is given by:

(4) $$M=\prod_{k=2}^{N}M_{N}=\left(\begin{array}{cc} M_{11} & M_{12}\\ M_{21} & M_{22} \end{array}\right)$$

where

(5) $$M_{N}=\left[\begin{array}{cc} \cos\beta_{N} & \frac{-i\sin\beta_{N}}{q_{N}}\\ -iq_{N}\sin\beta_{N} & \cos\beta_{N} \end{array}\right]$$

with

(6) $$q_{N}=\sqrt{\frac{\mu_{N}}{\varepsilon_{N}}}\cos\theta_{N}=\frac{{\left(\varepsilon_{N}-\varepsilon_{1}\sin^{2}\theta_{1}\right)}^{1/2}}{\varepsilon_{N}}$$

(7) $$\beta_{N}=\frac{2\pi }{\lambda }n_{N}d_{N}\cos\theta_{N}=\frac{2\pi d_{N}}{\lambda }{\left(\varepsilon_{N}-\varepsilon_{1}\sin^{2}\theta_{1}\right)}^{1/2}$$

where *λ* and *θ*_1_ represent the wavelength and angle of the incident light, respectively. *θ_N_*, $d_{N}$, and $\varepsilon_{N}$ are the angle of incident light, the thickness and dielectric constant of$\;N^{th}$ layer. According to the above equations, the reflection coefficient of the p-polarized incident light can be calculated as:

(8) $$r_{p}=\frac{\left(M_{11}+M_{12}q_{N}\right)q_{1}-\left(M_{21}+M_{22}q_{N}\right)}{\left(M_{11}+M_{12}q_{N}\right)q_{1}+\left(M_{21}+M_{22}q_{N}\right)}$$

Reflectance (Rp) for p-polarized incident light is given by:

(9) $$R_{p}={\left|r_{p}\right|}^{2}$$

The lateral position shift represents the penetration capacity of the evanescent wave, and the reference coefficient is determined by the standard eigenmatrix method [36]. The lateral position shift can be expressed as:

(10) ΔL=−λ2π|rp|2(Re(rp)dIm(rp)dθ−Im(rp)dRe(rp)dθ)

The performance of the proposed SPR sensor was derived based on the analytical algorithm of the transfer matrix method by using MATLAB software.

### 2.3. Performance Evaluation Formula

The crucial parameters that characterize the performance of the SPR sensors are sensitivity (S), FWHM, and figure of merit (FOM). The sensitivity represents the response of the SPR sensor to changes in the sensing medium and is represented by the following equation [37]:

(11) $$S=\frac{\Delta \theta }{\Delta n}$$

Δ*n* represents the change of the sensing medium (∆*n* = 0.005). Δ*θ* represents the change in resonance due to the change of the sensing medium. FWHM is the average angular variation corresponding to the maximum and minimum values of the SPR curve, reflecting the detection accuracy of the SPR sensor. To better understand the sensing performance of the SPR sensors, another intelligent scale of measurement called figure of merit (FOM) is applied to utilize the sensitivity and detection accuracy of the sensor and is represented by the following equation [37]:

(12) $$FOM=\frac{S}{FWHM}$$

## 3. Results and Discussion

**Figure 2:**
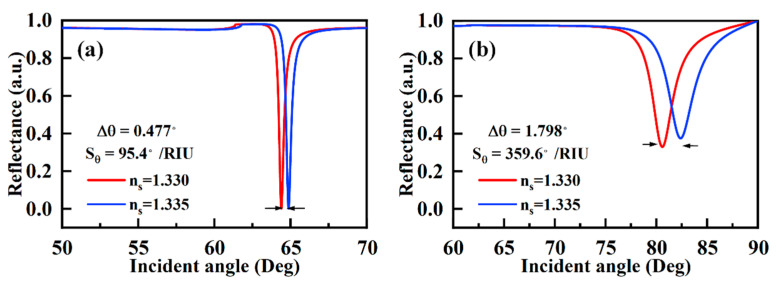
Reflectance variation under different incident angle for (**a**) SPR sensor with 40 nm Al film, (**b**) SPR sensor with the bimetallic layer of 40 nm Al and 22 nm Ni.

The simulation and optimization of the SPR sensor with the bimetallic layer are carried out by using TMM. Here, we first simulate the sensing performance of the SPR sensor comprised of a single metal layer. The simulation results of the SPR sensor with 40 nm Al layer are shown in Figure 2a, and the sensitivity is calculated to be 95.4 °/RIU, which is lower than the similar structure of Au and Ag [6,38]. Meanwhile, a thickness of 22 nm Ni film on the 40 nm Al layer has been proven to improve the sensitivity to 359.6 °/RIU as shown in Figure 2b. In addition to the previously reported function of Al–Au bimetallic layers to protect Al from oxidation, Al–Ni bimetallic layers have been shown to improve the sensitivity [39].

**Figure 3:**
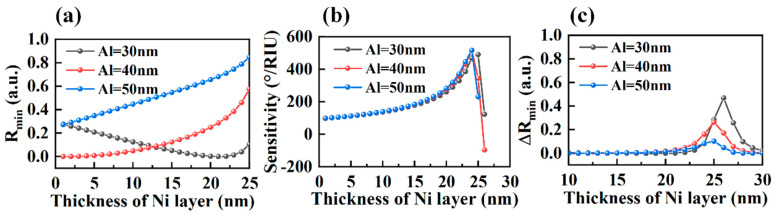
(**a**) The minimum reflectance of the bimetallic layer SPR sensor with various thicknesses of Al thin film (i.e., 30 nm, 40 nm, 50 nm) as a function of Ni thickness. (**b**) The sensitivity for different thicknesses of Al thin film (30 nm, 40 nm, 50 nm) at a fixed refractive index change in Δn = 0.005. (**c**) The difference of the minimum reflectance under the refractive indices of 1.335 and 1.330.

To further improve the performance of the SPR sensor based on the bimetallic film, the thickness of each layer should be optimized. The metal film plays a vital role in SPR sensing due to its significant influence on electric field enhancement. The sensitivity and minimum reflectance (R_min_) of the bimetallic SPR sensor for different thickness combinations of Al and Ni are shown in Figure 3. R_min_ is a reliable parameter to reflect the response of SPP, and the small R_min_ indicates that most of the incident light energy is transferred to SPP [40]. However, the sensitivity is not simply inversely proportional to R_min_ as shown in Figure 3a,b.

In order to reasonably evaluate the sensitivity of SPR, the difference of R_min_ under two refractive indices of external media is introduced in Figure 3c. The calculated results show that the sensitivity of the structure decreases when R_min_ reaches its maximum value. As shown in Figure 3a,b, the thickness variation of the Al layer has little effect on the maximum sensitivity, but on the contrary has a significant effect on the R_min_. Figure 3 shows that 40 nm aluminum is a better choice due to maximum sensitivity and small R_min_. In this structure, the SPR sensor based on Al(40 nm)–Ni(24 nm) achieves a high sensitivity of 517 °/RIU.

**Figure 4:**
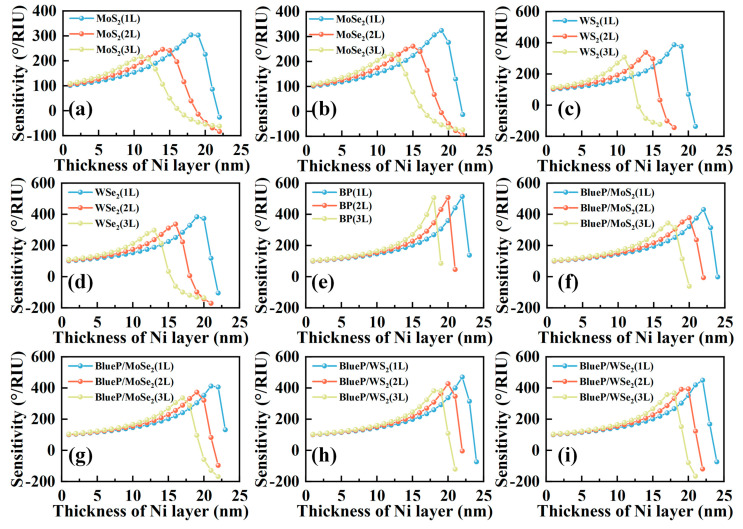
The sensitivity of SPR sensors with different 2D materials: (**a**) MoS_2_, (**b**) MoSe_2_, (**c**) WS_2_, (**d**) WSe_2_, (**e**) BP, (**f**) BlueP/MoS_2_, (**g**) BlueP/MoSe_2_, (**h**) BlueP/WS_2_, (**i**) BlueP/WSe_2_.

An additional 2D material can be added to the Al–Ni bimetallic layer for better biocompatibility and enriched functional groups. The optimized sensitivities for SPR sensors with different layers of 2D material on Al(40 nm)–Ni bimetallic film are shown in Figure 4a–i. When the thickness of Ni layer is low, the sensitivity of the sensor with multiple layers of 2D material, i.e., bilayer or trilayer, is higher than the sensor covered with a monolayer of 2D material. That can be explained by the effect of 2D material on the confinement of incident light for SPP excitation, and the contribution of light confinement on sensitivity improvement is higher than the light absorption loss caused by 2D material. With the increased thickness of the Ni layer, the sensor covered with trilayer of 2D material first reaches the maximal value of sensitivity, followed by the sensors with bilayer and monolayer 2D material. However, monolayer 2D material (blue line) on the bimetallic layer can achieve a higher value of maximum sensitivity than that of the bilayer (red line) and trilayer 2D material (yellow line) on the bimetallic layer. In addition, we also calculate the maximum sensitivities for the SPR sensors with even more layers of 2D material on the bimetallic layer which are collected in Figure S1, and the trend is consistent with the results shown in Figure 4. The parameters of the sensor obtaining the best performance are listed in Table 2. We can find that the highest sensitivities are 514.0 °/RIU, 303.8 °/RIU, 324.4 °/RIU, 387.6 °/RIU, and 383.6 °/RIU for the structures of Al–Ni bimetallic layer with monolayer BP, monolayer MoS_2_, monolayer WS_2_, monolayer MoSe_2_, and monolayer WSe_2_, respectively. After comparing the complex refractive index of TDMC and BP with similar imaginary part, 2D materials with large real part, such as MoS_2_ and WS_2_, exhibit a smaller FWHM than 2D materials with small real part, such as MoSe_2_ and WSe_2_. This means that 2D materials with large real part of the refractive index can enhance the local confinement of SPP, resulting in a reduction in FWHM and an increase in sensitivity. Meanwhile, the imaginary part of the refractive index has a significant effect on the sensitivity of 2D materials. BP has the lowest real and imaginary parts of refractive index, but the sensor based on BP performs with the highest sensitivity. For this phenomenon, we believe that the effect of light energy loss caused by 2D material is greater than its contribution to the surface electric field enhancement in our structure. This phenomenon generally exists in SPR sensors adopting 2D materials, which is especially manifested when discussing the effect of 2D material layers on sensitivity.

To verify this assumption, a class of BlueP/TDMC, whose real and imaginary parts of the refractive index are lower than TMDC, is selected for the simulation. BlueP is also an excellent 2D material suitable for SPR sensors, which has low loss, tunable direct band gap, high carrier density, and large specific surface area. In addition, since BlueP and TMDC have the same hexagonal crystal structure, they can be fabricated in a hybrid structure by stacking to prevent the oxidation of BlueP [30]. Simulation results show that the sensitivity of the SPR sensor of the bimetallic layer attached with BlueP/TMDC is about 15% higher than the one attached with TMDC.

**Figure 5:**
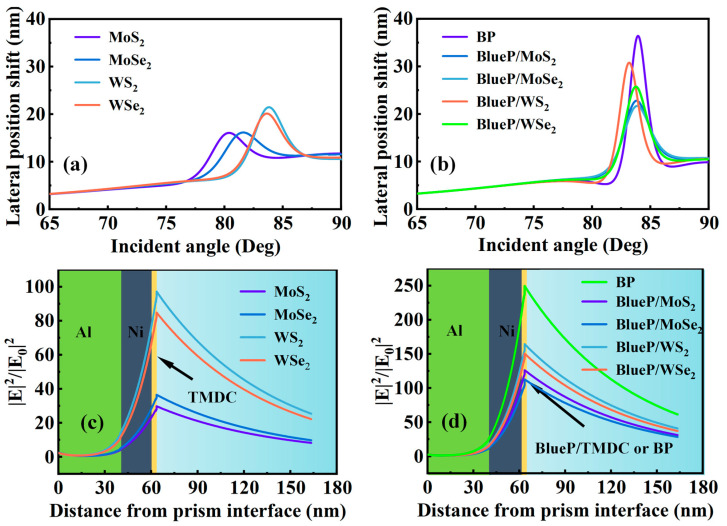
The lateral position shift of the proposed SPR sensor with (**a**) TMDC, and (**b**) BP and BlueP/TMDC. The electric field distributions of the proposed SPR sensor with (**c**) TMDC, and (**d**) BP and BlueP/TMDC.

It is also noticed that the sensitivity of the SPR sensor attaching with BlueP/MoS_2_ is higher than that of the SPR sensor attaching with WS_2_; however, this is difficult to explain through the direct comparison of the refractive indices, as the two materials have similar imaginary parts, and the real part of BlueP/MoS_2_ is smaller than that of WS_2_. To clarify the mechanism, we need to understand the effect of 2D materials on the overall sensor structure. Generally, the presence of the 2D material layer will lead to the reflection at the interface of the 2D material/Ni layer, the absorption of 2D material itself, and the reflection at the outermost interface. Since the reflection at the interfaces of sensing medium/Ni and sensing medium/2D material are both close to the total internal reflection, the influence of 2D material on the reflection of the outmost interface is negligible. Considering the rest of the two factors, we believe that the utilization of incident light energy is the major influencing factor which needs to consider both the real and imaginary parts of the refractive index. In order to prove this view, the lateral position shift at the interface of 2D material/sensing medium is introduced and the calculation results are shown in Figure 5a,b. The sensitivity and lateral position shift for all SPR structures with different 2D material are provided in Table S1. The interesting result is that the SPR structures with large lateral position shift will exhibit high sensitivity at similar resonance angles. We think its physical meaning can be explained in that the lateral position shift represents the penetrating ability of the evanescent wave. The larger the lateral position shift, the more energy of incident light reaches the 2D material/sensing medium interface for achieving large interaction distance of the evanescent wave with the sensing medium.

**Figure 6:**
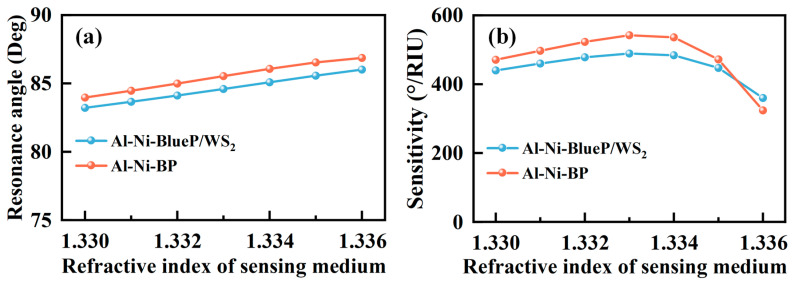
(**a**) Resonance angles corresponding to the different refractive index of sensing media for the SPR sensors with Al(40 nm)–Ni(22 nm)–BlueP/WS_2_(1 L) and Al(40 nm)–Ni(22 nm)–BP(1 L), (**b**) Sensitivity of SPR sensors under the refractive index of the sensing medium varying from 1.330–1.336 (∆n = 0.001).

The electric field distributions from the prism to the external medium have been calculated for the SPR sensors with TMDC and BlueP/TMDC and are shown in Figure 5c,d, respectively. The electric field intensity along the structure is another intuitive representation of the energy distribution, and this parameter also takes both real and imaginary parts of the refractive index into account. The simulation result completely meets our expectations. The SPR structure with high sensitivity has a large lateral position shift and large electric field intensity. The electric field intensity of the SPR sensor using BlueP/TMDC as the outmost layer is much higher than that of the SPR sensor using TMDC. This result also proves that BP and BlueP/TMDC are more efficient in the utilization of incident light energy in our proposed structure. The sensing ranges of the sensors are also presented in Figure 6a,b. In the refractive index sensing range of 1.330–1.335, the proposed SPR sensors ensure a highly sensitive and flat response, which has been demonstrated to exhibit average sensitivity of 466 °/RIU for Al(40 nm)–Ni(22 nm)–BlueP/WS_2_(1 L) structure and 507 °/RIU for Al(40 nm)–Ni(22 nm)–BP(1 L) structure. Meanwhile, the maximum sensitivities are 489 °/RIU for Al(40 nm)–Ni(22 nm)–BlueP/WS_2_(1 L) structure and 542 °/RIU for Al(40 nm)–Ni(22 nm)–BP(1 L) structure when the external refractive index is 1.333. In addition, the sensitivities of SPR sensors based on Ni–2D material with SF10 prism have also been calculated and the results are shown in Figures S2 and S3 and Table S2, in which it can be observed that the sensitivity of Ni–2D material is difficult to surpass the result of Al/Ni–2D material. Finally, the sensitivities of the proposed sensors at the refractive index of 1.330 are compared with those previously stated by researchers with different configurations and listed in Table 3. The feasibility of the experimental method, including SPR sensors preparation method and experimental system diagram (Figure S4), is also provided in supplementary materials.

## 4. Conclusions

In this study, a novel SPR biosensor based on the Kretschmann configuration with high sensitivity by using Al–Ni bimetallic layer and 2D material is presented. The thicknesses of Al and Ni films are optimized to achieve suitable R_min_ and maximum sensitivity. The sensitivities of Al–Ni bimetallic layer covered with different 2D materials are also compared, and results show that the angular sensitivity can reach as high as 489 °/RIU for Al(40 nm)–Ni(22 nm)–BlueP/WS_2_(1 L) structure and 542 °/RIU for Al(40 nm)–Ni(22 nm)–BP(1 L) structure. Meanwhile, the proposed SPR sensors ensure a highly sensitive and flat response in the refractive index range of 1.330–1.335. In addition, both the real and imaginary parts of the refractive index of 2D materials have been demonstrated to exhibit a significant impact on the maximum sensitivity of the SPR sensor, which is interpreted by using the parameters of lateral position shift for the optimization of 2D materials on SPR sensors. Despite the consistency of lateral position shift and angle sensitivity demonstrated in our theoretical model, further study on the SPR sensor with different structures is still necessary to theoretically and experimentally verify the relationship of the two parameters. We hope that the study presented in this paper will be beneficial for the design and application of 2D material-based SPR sensors.

## Figures and Tables

**Table 1:** Thickness and refractive index of 2D materials at the wavelength of 633 nm.

Material	Thickness of Monolayer (nm)	Refractive Index
MoS_2_	0.65	5.08 + 1.1723 i
MoSe_2_	0.70	4.62 + 1.0063 i
WS_2_	0.80	4.9 + 0.3124 i
WSe_2_	0.70	4.55 + 0.4332 i
BP	0.53	3.5 + 0.01 i
BlueP/MoS_2_	0.75	2.81 + 0.32 i
BlueP/MoSe_2_	0.78	2.77 + 0.35 i
BlueP/WS_2_	0.75	2.48 + 0.17 i
BlueP/WSe_2_	0.78	2.69 + 0.22 i

**Table 2:** The highest angular sensitivity for the SPR sensors based on Al(40 nm)–Ni–2D material at the refractive index of 1.330.

Material	Optimized Layers (L)	Thickness of Ni (nm)	Sensitivity (°/RIU)	FWHM (Deg)	FOM
BP	1	22	514.0	3.890	132.1337
MoS_2_	1	18	303.8	6.941	43.7689
MoSe_2_	1	19	324.4	7.003	46.3230
WS_2_	1	18	387.6	4.819	80.4316
WSe_2_	1	19	383.6	4.982	76.9972
BlueP/MoS_2_	1	22	420.4	5.111	82.2540
BlueP/MoSe_2_	1	21	412.2	4.440	92.8378
BlueP/WS_2_	1	22	470.2	4.187	112.3000
BlueP/WSe_2_	1	22	449.8	4.714	95.4179

**Table 3:** Angle sensitivity comparison of SPR sensors.

Reference	Multi-Layer Structure	Angular Sensitivity@1.330 (°/RIU)	Maximum Angular Sensitivity (°/RIU)	Average Angular Sensitivity (°/RIU)
[41]	Au–MoS_2_–Ni–Graphene	229	286	238@1.33~1.35
[11]	Ag–Au–BaTiO_3_–Graphene	294	294	283@1.332~1.346
[31]	Ag–WS_2_–Ni–Graphene	243.3	-	-
[16]	Cu–Ni–WS_2_	480.0	-	-
[12]	WS_2_–Al–WS_2_–Graphene	315.5	-	-
This Work	Al–Ni–BlueP/WS_2_	470.2	489	466@1.330~1.335
This Work	Al–Ni–BP	514.0	542	507@1.330~1.335

## Data Availability

The data are available from the corresponding author Haifei Lu, upon reasonable request.

## Supplementary Materials

The following supporting information can be downloaded at: https://www.mdpi.com/article/10.3390/s23031714/s1, Figure S1: Sensitivity as a function with respect number of (a) TMDC (b) BP and BlueP/TMDC; Table S1: The highest surface electric field intensity, lateral position shift and sensitivity for the Al(40 nm)-Ni-2D materials of SPR sensors; Figure S2: The sensitivity of the SPR sensors based on Ni-2D materials. (a) MoS2 (b) MoSe2 (c) WS2 (d) WSe2 (e) BP (f) BlueP/MoS2 (g) BlueP/MoSe2 (h) BlueP/WS2 (i) BlueP/WSe2; Figure S3: Sensitivity as a function with respect number of (a) TMDC (b) BP and BlueP/TMDC; Table S2: The highest angular sensitivity for the SPR sensors based on Ni-2D material at the refractive index of 1.330; Figure S4: Schematic diagram of the experimental setup for SPR sensor. References [42,43,44] are cited in the supplementary materials.
