# Supplementary material for: Sensitivity Enhancement of 2D Material-Based Surface Plasmon Resonance Sensor with an Al–Ni Bimetallic Structure
Source: Sensors (Basel). 2023 Feb 3;23(3):1714. doi: 10.3390/s23031714 (PMC9921370; doi:10.3390/s23031714)
Supplement: Supplementary file 1 [file sensors-23-01714-s001.zip › sensors-2190753-supplementary.pdf]

Supplementary Materials

Sensitivity Enhancement of 2D Material-Based Surface Plasmon Resonance Sensor with an Al–Ni Bimetallic Structure

Miaosen Hu [1], Min Li [1], Ming-Yu Li [1,2], Xiaoyan Wen [1], Shuo Deng [1], Sisi Liu [1] and Haifei Lu [1,2,*]

1 School of Science, Wuhan University of Technology, Wuhan 430070, China
2 Yangtzi Delta Region Institute of University of Electronic Science and Technology of China, Huzhou 313098, China
* Correspondence: haifeilv@whut.edu.cn

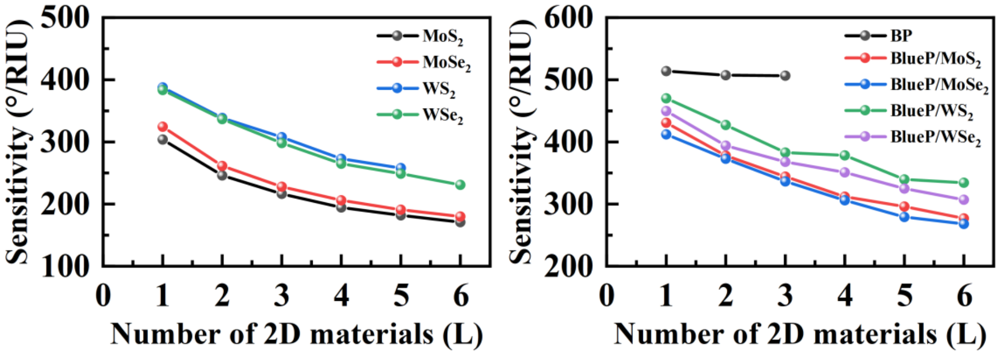

Figure S1. Sensitivity as a function with respect number of (a) TMDC (b) BP and BlueP/TMDC.

Table S1 The highest surface electric field intensity, lateral position shift and sensitivity for the Al(40 nm)-Ni-2D materials of SPR sensors.

Material	Sensitivity (°/RIU)	surface electric field intensity ($|E|^2/|E_0|^2$)	lateral position shift (nm)
MoS_2	303.8	29.75	16.05
$MoSe_2$	324.4	36.49	16.14
WS_2	387.6	97.15	21.45
WSe_2	383.6	84.82	20.12
BlueP/MoS_2	420.4	126.11	21.68
BlueP/$MoSe_2$	412.2	112.56	22.78
BlueP/WS_2	470.2	164.31	30.77
BlueP/WSe_2	449.8	150.40	25.73
BP	514.0	249.50	36.40

The SPR structure of BK7-Ni-2D material is difficult to excite SPP due to the low refractive index of BK7 prism which has been replaced by SF10 prism [1]. The sensitivity of SPR sensors based on Ni-2D material are shown in Figure S3(a-i) and the maximum sensitivity as a function with 2D material layer are shown in Figure S4(a) and S4(b). The

structure of Ni(58nm)-BP(4L) exhibits a maximum sensitivity of 401.4 °/RIU, which is lower than the sensitivity of the sensors based on Al-Ni-BP and Al-Ni-BlueP/WS_2. The

specific thickness and maximum sensitivity for SPR sensors with optimized thickness Ni and 2D material layer are listed in Table S2.

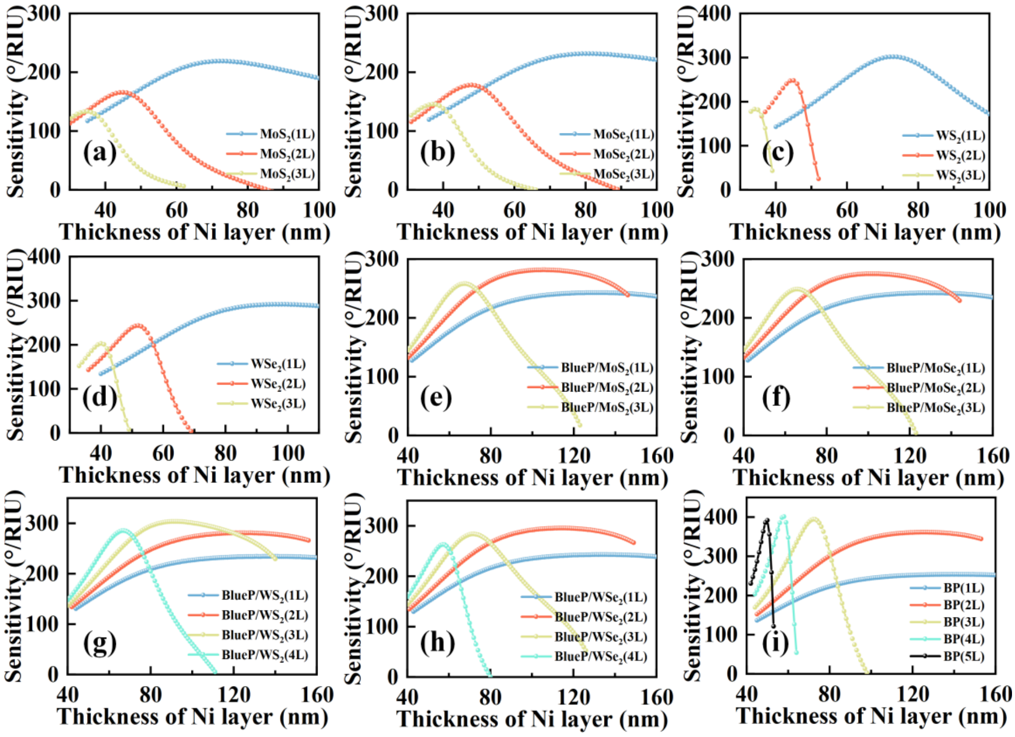

Figure S2. The sensitivity of the SPR sensors based on Ni-2D materials. (a) MoS_2 (b) $MoSe_2$ (c) WS_2 (d) WSe_2 (e) BP (f) BlueP/MoS_2 (g) BlueP/$MoSe_2$ (h) BlueP/WS_2 (i) BlueP/WSe_2.

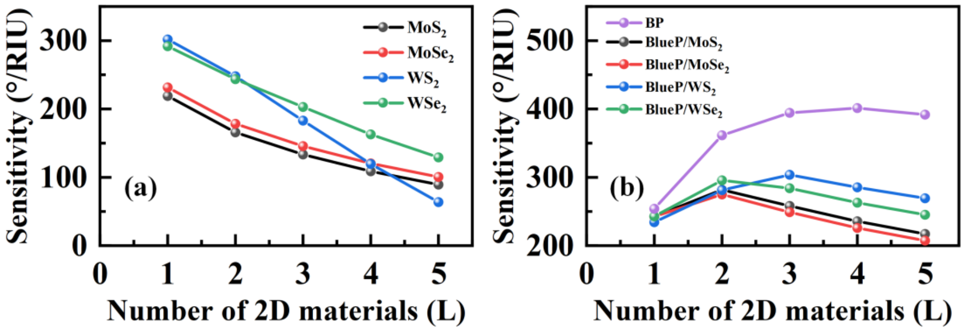

Figure S3. Sensitivity as a function with respect number of (a) TMDC (b) BP and BlueP/TMDC.

Table S2 The highest angular sensitivity for the SPR sensors based on Ni-2D material at the refractive index of 1.330.

Material	Optimized layers (L)	Thickness of Ni (nm)	Sensitivity (°/RIU)
BP	4	58	401.4
MoS_2	1	72	219
$MoSe_2$	1	80	231.6
WS_2	1	73	301.8
WSe_2	1	98	291.8
BlueP/MoS_2	2	105	281.8
BlueP/$MoSe_2$	2	100	275
BlueP/WS_2	3	90	303.8
BlueP/WSe_2	1	123	281.2

Figure S2 is the schematic diagram of experimental setup for the SPR sensor. The incident light is excited by a He-Ne laser with a wavelength of 633nm. Then, the incident beam passes through Beam shaping module and reaches the SPR sensor to excite the SPP. The reflected beam is collected by a photodetector and uploaded to a computer for data processing. For the preparation of SPR sensor, Al and Ni can be sequentially prepared on BK7 glass substrate by magnetron sputtering in vacuum chamber to avoid the oxidation of Al layer. The two-dimensional material can be either synthesized using chemical vapor deposition method [2] or mechanically transferred on the bimetallic layer. The preparation of BP and BlueP can be found in previous publications [3], and the transferring of BlueP or TMDC has been also broadly reported [4].

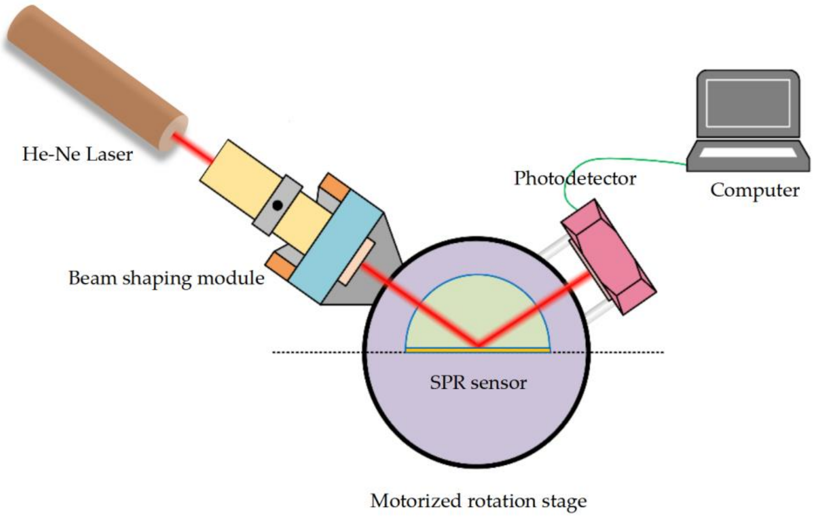

Figure S4. Schematic diagram of the experimental setup for SPR sensor.

References

1. Shivangani; Alotaibi, M. F.; Al-Hadeethi, Y.; Lohia, P.; Singh, S.; Dwivedi, D. K.; Umar, A.; Alzayed, H. M.; Algadi, H.; Baskoutas, S., Numerical Study to Enhance the Sensitivity of a Surface Plasmon Resonance Sensor with BlueP/WS(2)-Covered Al(2)O(3)-Nickel Nanofilms. *Nanomaterials (Basel)* **2022,** 12, (13).
2. Deng Y, L. Z., Conrad NJ, Liu H, Gong Y, Najmaei S, Ajayan PM, Lou J, Xu X, Ye PD. , Black phosphorus-monolayer MoS2 van der Waals heterojunction p-n diode. **2014,** 8, (8), 8292-9.
3. Mao, N.; Tang, J.; Xie, L.; Wu, J.; Han, B.; Lin, J.; Deng, S.; Ji, W.; Xu, H.; Liu, K.; Tong, L.; Zhang, J., Optical Anisotropy of Black Phosphorus in the Visible Regime. *J Am Chem Soc* **2016,** 138, (1), 300-5.
4. Liu, Y.; Weiss, N. O.; Duan, X.; Cheng, H.-C.; Huang, Y.; Duan, X., Van der Waals heterostructures and devices. *Nature Reviews Materials* **2016,** 1, (9).
